# Impact of hormonal treatments for endometriosis on the reproductive microbiome: a systematic review

**DOI:** 10.3389/fmicb.2026.1755725

**Published:** 2026-02-12

**Authors:** Stefania Luppi, Ghergana Alexandrova Topouzova, Giuseppina Campisciano, Elena Giolo, Teresa Bulfone, Francesca Rossi, Gabriella Zito, Giuseppe Ricci, Manola Comar, Eva Andreuzzi

**Affiliations:** 1 Physiopathology of Human Reproduction, Institute for Maternal and Child Health, IRCCS “Burlo Garofolo”, Trieste, Italy; 2 Department of Medicine, Surgery and Health Sciences, University of Trieste, Trieste, Italy; 3 Advanced Translational Microbiology, Institute for Maternal and Child Health, IRCCS “Burlo Garofolo”, Trieste, Italy; 4 Maternal-Neonatal Department, Institute for Maternal and Child Health, IRCCS “Burlo Garofolo”, Trieste, Italy

**Keywords:** adult and young patients, endometrial microbiome, endometriosis, hormonal therapy, reproductive health, vaginal microbiota

## Abstract

**Introduction:**

The reproductive microbiome plays a key role in disease progression and fertility in women with endometriosis. Vaginal and endometrial dysbiosis has been increasingly linked to inflammation, impaired reproductive outcomes, and symptom severity. Although estro-progestins, progestins, and GnRH agonists are widely used, their impact on microbial communities remains poorly understood, highlighting the need to clarify microbiome–therapy interactions. This systematic review aims to comprehensively synthesize current evidence on how hormonal therapies influence the reproductive microbial environment and to offer insights for optimizing clinical management of endometriosis.

**Methods:**

Literature screening and data extraction followed PRISMA guidelines using PubMed, Scopus, and Google Scholar. The search combined terms on endometriosis, hormonal therapy, and reproductive microbiome. Non-English studies, reviews, and those without original data were excluded. Risk of bias was assessed with ROBINS-I-V2, and microbial composition and diversity were analyzed and synthesized qualitatively.

**Results:**

The literature search retrieved 577 publications, of which 6 met eligibility criteria and were analyzed. The evidence collected through sequencing or culture-based methods suggested that the use of hormonal therapies to treat endometriosis may impact both vaginal and endometrial microbiome, favoring the colonization of bacterial species associated with infertility. GnRHa resulted to foster the dominance of potentially pathogenic bacteria, as *Gardnerella* and *Streptococcaceae*, in the endometrium, and supporting bacterial vaginosis by increasing intermediate flora (Nugent score 4–6). A similar effect on the vaginal environment has been reported upon the use of oral contraceptive pills, which was shown to prompt the increase of *Prevotella*, *Ureaplasma*, *Streptococcus anginosus* and *Streptococcus agalactiae*, among other pathogenic microbes, and to enhance the *Bacillota/Bacteroidota* ratio.

**Discussion:**

Despite affected by several limitations and heterogeneity of included studies, this review provides a preliminary overview of the possible pejorative effect of hormonal therapy on the reproductive microbiome of endometriosis patients. While further investigations are required to consolidate these findings, the observations raised offer a valuable basis for opening a discussion about improving management strategies for affected women. By highlighting confounding factors overlooked in the selected papers, the present work will also be functional to optimize the design of future studies.

**Systematic review registration:**

https://www.crd.york.ac.uk/PROSPERO/view/CRD420251042858, identifier PROSPERO (CRD420251042858)

## 1 Introduction

Female reproductive tract harbors a distinct and characteristic microbial community, accounting for approximately 9% of the total bacterial population in the female organism (Punzón-Jiménez and Labarta, 2021). It is now well established that the human microbiome is the result of a complex network of microorganisms distributed in various parts of the organism, whose interaction determines the state of health and disease (Martínez et al., 2021). The female reproductive tract microbiota represents a continuum in the genital tract (Chen et al., 2017) and provides several benefits through a series of physiological functions throughout woman life such as protection against infections, immune system and inflammation modulation, fertility support and pregnancy maintenance (Agostinis et al., 2019; Auriemma et al., 2021; Zheng et al., 2025). It is susceptible to various factors as hormones and lifestyle, which can induce alterations of the microbial environment (i.e., dysbiosis), such as a decrease in beneficial *Lactobacilli* and in microbial diversity (*α*-diversity) along with an increase in potentially pathogenic or pro-inflammatory bacterial species (Kobayashi, 2023). Current evidences have shown that quantitative and qualitative imbalances between pathogenic and non-pathogenic commensal bacteria is closely associated with hormonal and immune system fluctuations, key drivers of gynecological diseases as endometriosis (Ata et al., 2019; Doroftei et al., 2021; Jiang et al., 2021; Zhu et al., 2022).

Endometriosis represents a multifactorial, estrogen-dependent disease, that affects 10% of women of childbearing age worldwide, with symptoms often beginning in adolescence or young age (8–25 years old) (Oliveira et al., 2025). It is characterized by ectopic growth of endometrial tissue which can be confined to the genital tract or may spread throughout the peritoneal cavity or other locations outside the abdomen, however to date its pathogenesis remains still incompletely understood (As-Sanie et al., 2025). Endometriosis has a significantly impact on quality of life as it can entail important consequences for affected women, including severe primary dysmenorrhea, dyspareunia, chronic pelvic pain and infertility (Johnson et al., 2017; Laganà et al., 2019). Recent studies evidenced a possible correlation between endometriosis and dysbiosis, highlighting that endometriosis significantly associates with the absence of *Lactobacilli* (Findeklee et al., 2023) and with over-representation of *Streptococcus* in genito-urinary apparatus (Miyashira et al., 2022). These alterations can contribute to inflammatory and immunological processes, and exacerbate hormonal dysregulation and infertility (Borelli et al., 2019; Campisciano et al., 2020; Leonardi et al., 2020; Colonetti et al., 2023; Toffoli et al., 2025). Nonetheless, the scientific community still debates whether dysbiosis can contribute to the development of endometriosis or vice versa. Some authors hypothesize that the reciprocal detrimental influence between endometriosis and dysbiosis may establish a vicious cycle that further contributes to disease progression (Wang et al., 2025). In contrast, a recent systematic review conducted by Facciotti et al. (2025) revealed a significant heterogeneity among both women with and without endometriosis, and an absence of sufficient data to demonstrate a difference in the microbial profile of the two groups.

Nevertheless, in their metanalysis Haahr et al. evidenced a very low prevalence of endometriosis among patients with bacterial vaginosis (BV), which is a microbial dysbiosis characterized by a shift in the normal *Lactobacillus*-dominant vaginal microbiota to a marked increase of heterogeneous anaerobic and facultative anaerobic species such as *Gardnerella vaginalis, Prevotella bivia, Atopobium vaginae, Mobiluncus curtisii* and *Mycoplasma hominis* (Haahr et al., 2019; Segui-Perez et al., 2025). To explain this finding, the authors hypothesized that, in endometriosis patients, hormonal treatment may favor a healthy microbial colonization. Indeed, it has been demonstrated that exposure to estrogens-progestins combined oral contraceptives (COCs) may positively influence gynecological health through an increase in healthy *Lactobacilli* and a decrease in BV-associated bacterial taxa (Brooks et al., 2017). In contrast, other authors stated that no strong evidence exists regarding the use of oral contraceptive pills and alterations of the vaginal microbiome (Balle et al., 2023). Thus, a unanimous agreement on this topic is lacking and still debated among the scientific community.

**Figure 1:**
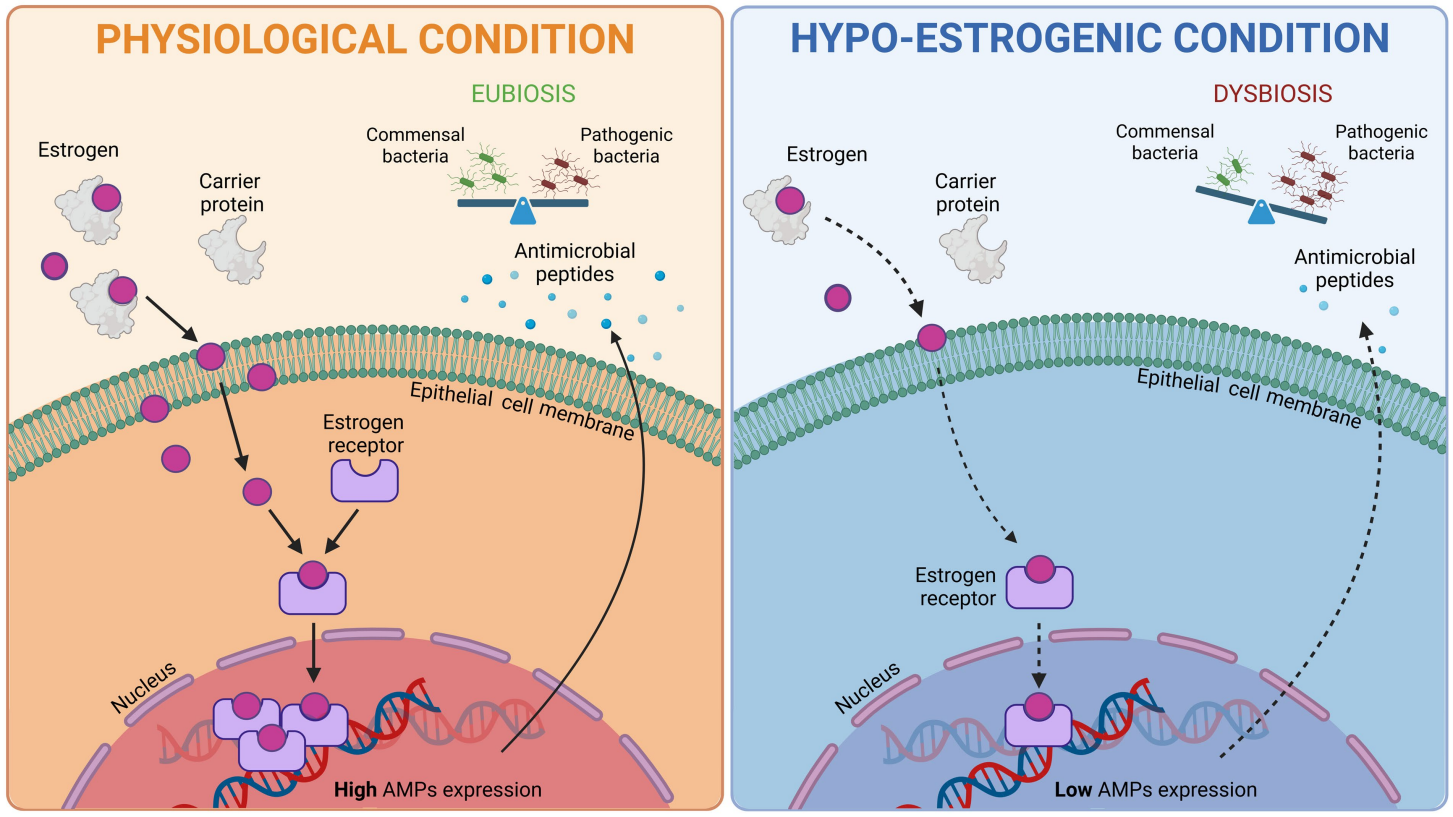
Schematic representation of the influence of estrogen on the release of anti-microbial peptides (AMPs) by epithelial cells. Under physiological conditions estrogen uptake and receptor activation trigger AMPs expression and secretion, thus favoring a balanced microbiota (eubiosis). Hormonal therapy establishes a hypo-estrogenic environment which results in a decreased AMPs secretion and a shift toward pathogenic bacteria colonization (dysbiosis). Created in https://BioRender.com.

The hormonal treatment for endometriosis aims to block menstrual cycle by inhibiting the hypothalamus-pituitary-ovary axis, and almost eliminating the production of endogenous estrogens, causing consequently amenorrhea. The hypo-estrogenic environment that is created hinders the progression of endometriosis implants. Multiple international guidelines state that first-line hormonal treatment for women diagnosed with endometriosis who are not seeking immediate pregnancy includes COCs or progestins-only medications, whereas second-line hormonal treatment includes gonadotropin-releasing hormone (GnRH) agonists and antagonists (Committee on Practice Bulletins—Gynecology, 2010; Update Information, 2017; Becker et al., 2022; As-Sanie et al., 2025). Prolonged therapy with GnRH agonists (GnRHa) is generally associated with administration of COCs (add-back therapy) after a few months, to prevent hypo-estrogenic side effects, including bone mineral density loss and vasomotor symptoms (Surrey, 2022). Recent observations indicate that the use of GnRHa and progestins is more effective rather than COCs in reducing disease-associated pain (Vannuccini et al., 2022). Further research is needed to fully evaluate the role of different hormonal treatments in the management of endometriosis. Importantly, sex steroids, especially estrogens, are known to regulate the release of anti-microbial peptides by epithelial cells both in the gut and in the genito-urinary tract, significantly influencing microbial colonization (Figure 1; Vodstrcil et al., 2013; Medina-Estrada et al., 2018; Ratten et al., 2021), which in turn affects estrogen metabolism (Li Z. et al., 2025). Despite this evidence, the impact of hormonal therapies on the reproductive microbiome of women with endometriosis remains poorly understood.

The aim of this review is to systematically examine the updated literature regarding the effect of hormonal therapy to treat endometriosis on the microbial profiles of the female reproductive tract, with the purpose to unveil potential associations and provide evidence to assist clinicians in a better management of patients.

## 2 Methods

### 2.1 Protocol and registration

The systematic review was designed based on the Preferred Reporting Ideas for Systematic Review and Meta-analyses (PRISMA) (Page et al., 2021) systematic review checklist and was registered on PROSPERO (Date of registration: July 22nd 2025, ID: CRD420251042858, review protocol link: https://www.crd.york.ac.uk/PROSPERO/view/CRD420251042858).

### 2.2 Search strategy—eligibility criteria, information sources, and search terms

Original research articles written in English and published between 1st January 2000 and 31st December 2025 were eligible for inclusion. We included studies reporting any relation between hormonal therapy to treat endometriosis and the endometrial, cervical and vaginal microbial profile. Studies regarding women diagnosed with endometriosis based on clinical evaluation, imaging or surgical confirmation (e.g., laparoscopy) and under hormonal therapy were considered. Intervention included the administration of: GnRH analogs, estrogen-progestin pills, only progesterone pills. Whereas, studies based on the use of intra-uterine devices were excluded. Control population included patients with endometriosis who did not receive hormonal treatments. Randomized and nonrandomized studies conducted in hospitals, clinics and universities worldwide were taken into consideration. Articles not published in English, whose full text was not available, letters to the editor, narrative and systematic reviews, preprint, guidelines, book chapters, authors’ replies, case reports, and poster presentations were excluded. Systematic reviews concerning the topic of the study have been examined to identify eligible articles which were not found by database searching.

To ensure a comprehensive retrieval of all the studies relative to endometriosis and the microbial profile of female reproductive tract, we chose to exploit three relevant and reliable databases: PubMed (MEDLINE), Scopus and Google Scholar. The combination of MeSH terms searched in the databases included “endometriosis,” “vaginal microbiome,” “endometrial flora,” “bacterial vaginosis,” “vaginal dysbiosis,” “GnRH agonist,” “progestin,” “combined oral contraceptive”, “oral hormonal therapy”. The detailed search terms for all databases are provided in Supplementary Table 1.

### 2.3 Study selection and data extraction

Duplicate articles were removed from the results of the literature search. Two independent authors screened the titles and abstracts of the remaining articles to ensure that the eligibility criteria were met. Any discrepancies between the authors were identified and discussed (with inputs from a third author if required). The remaining included articles were assessed by full-text screening by two independent authors, using the same eligibility criteria.

### 2.4 Assessment of risk of bias

Risk of bias assessment was carried out by using Version 2 of the Cochrane risk-of-bias tool for Non-randomized Studies of Interventions (ROBINS-I-V2) (Risk of Bias Tools, n.d.). Three reviewers (EA, SL and GC) independently assessed the studies for potential biases, including selection and reporting biases. The overall risk of bias is a synthesis of the three independent evaluations. Any discrepancies were resolved through discussion.

### 2.5 Data synthesis

The extracted data were synthesized according to the design and outcome measures of the included studies. Given the paucity of studies identified after selection, and the limited number of patients analyzed in each study, a meta-analysis could not be performed. The main outcomes evaluated for each study were: microbiota composition and relative abundance of bacterial communities in cervical, vaginal and endometrial samples; *α*-diversity and *β*-diversity; imbalances or disruptions in the normal microbiota, including a decrease in beneficial bacteria or an increase in harmful microbes (e.g., *Lactobacillus* dominance and specific taxa colonization); Nugent score for bacterial vaginosis. The findings were synthesized in tables by using a qualitative narrative approach.

## 3 Results

### 3.1 Study selection

**Figure 2:**
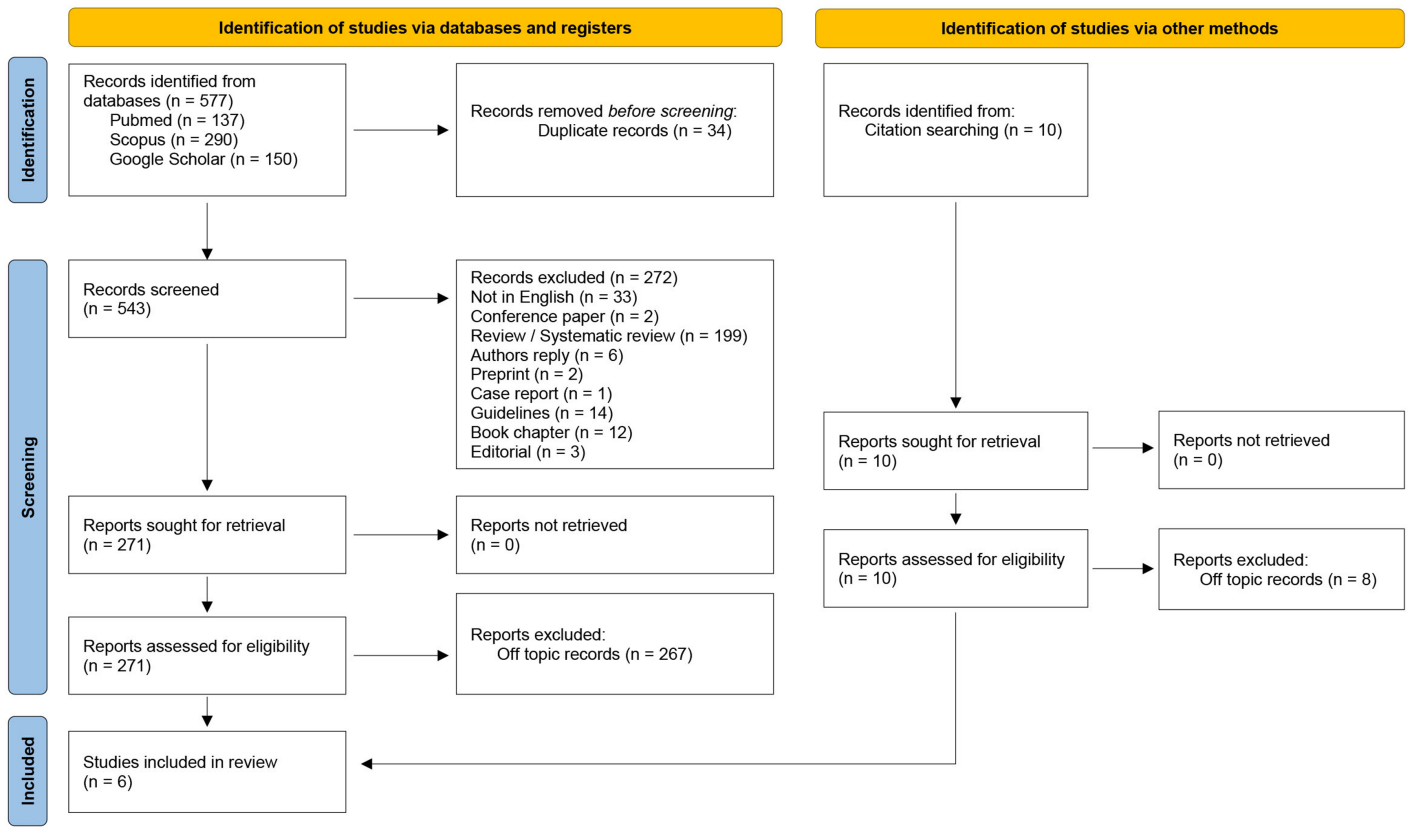
PRISMA flow diagram of the studies’ screening and selection.

The systematic search identified a total of 577 articles: 137 articles were available in PubMed, 290 studies in Scopus, and 150 in Google Scholar. Among those, 34 were duplicates and 272 articles were excluded since the publication type did not meet the eligibility criteria (non-English articles, reply to the editor, narrative and systematic reviews, preprint, guidelines, conference abstracts, case reports, book chapters). Two independent authors screened a total of 271 articles for their relevance in the topic by assessing the title, abstract or full-text. Among the 271 articles, 267 studies were excluded since unrelated to the effect of hormonal therapy on the microbial profile of endometriosis patients. During the screening process, upon the evaluation of systematic reviews concerning the object of the study, 2 articles not identified by the database searches but relevant for the present review were found and added manually to the list. As a result, 6 articles were included and analyzed in this systematic review (Figure 2).

### 3.2 Study characteristics

The literature search was conducted over the period from 1st January 2000 to 31st December 2025. The 6 identified studies included in the analysis were all published after 2014. This is probably due to the fact that the importance of the microbiome in reproductive biology gained more attention in the last decade. Furthermore, the technological advances of recent years, particularly concerning next generation sequencing (NGS) methods, have enabled the characterization of the complete microbiota of the female reproductive tract with greater precision and cost-effectiveness.

Among the selected papers, three investigations were conducted in Japan, two in the USA and one in Australia (Table 1). Despite the inclusion criteria for literature searching not specified a range of age, all the studies included women spanning from 18 to 51 years old. In the papers from Khan et al. (2014), Khan et al. (2016), Wee et al. (2018), and Khan et al. (2021), all patients analyzed had a regular menstrual cycle (28–32 days), whereas Do et al. (2024) and Le et al. (2021) did not provide this information.

**Table 1:** Overview of the characteristics of the studies included in the review.

Authors	Year	Country	Ethnicity	Design	Sample size (endometriosis patients)	Range of age (years)	Regular menstrual cycle (28–32 days)	Menstrual cycle phase (P/S/M/A)	Endometriosis staging
Khan et al. (2014)	2014	Japan	N/I	Prospective case–control	54 (15 HT, 39 NT)	21–51	Yes	P/S/M	laparoscopy, r-ASRM staging I, II, III e IV
Khan et al. (2016)	2016	Japan	N/I	Case–control	32 (16 HT, 16 NT)	21–47	Yes	HT: A; NT: P/S/M	laparoscopy, r-ASRM staging I, II, III e IV
Wee et al. (2018)	2018	Australia	N/I	Retrospective case–control	12 (9 HT, 3 NT)	35–45	N/I	P	N/I
Le et al. (2021)	2021	USA	Caucasian, Hispanic, unspecified	Case–control	20 (10 HT, 10 NT)	18–51	N/I	N/I	laparoscopy, r-ASRM staging I, II, III e IV
Khan et al. (2021)	2021	Japan	N/I	Prospective non-randomized observational study	32 (11 HT, 21 NT)	18–51	Yes	HT: A; NT: P/S/M	laparoscopy, r-ASRM staging I, II, III e IV
Do et al. (2024)	2024	USA	Caucasian, African American, Asian, unspecified	Case–control	33 (18 HT, 15 NT)	18–51	N/I	N/I	laparoscopy, I, II, III e IV

N/I, not indicated; HT, hormonal treatment; NT, not treated; P, proliferative phase; S, secretory phase; M, menstrual phase; A, amenorrhea.

In four papers out of six, endometriosis was diagnosed by laparoscopy and the staging of the disease based on the revised classification of the American Society of Reproductive Medicine (r-ASRM) (American Society for Reproductive Medicine, 1997). In one paper, endometriosis was assessed through laparoscopy, however the classification used for disease staging was not specified. The paper from Wee et al., on the contrary, based on the clinical history of the patients collected through a self-completed questionnaire, without considering the stage of the disease.

### 3.3 Risk of bias of included studies

**Figure 3:**
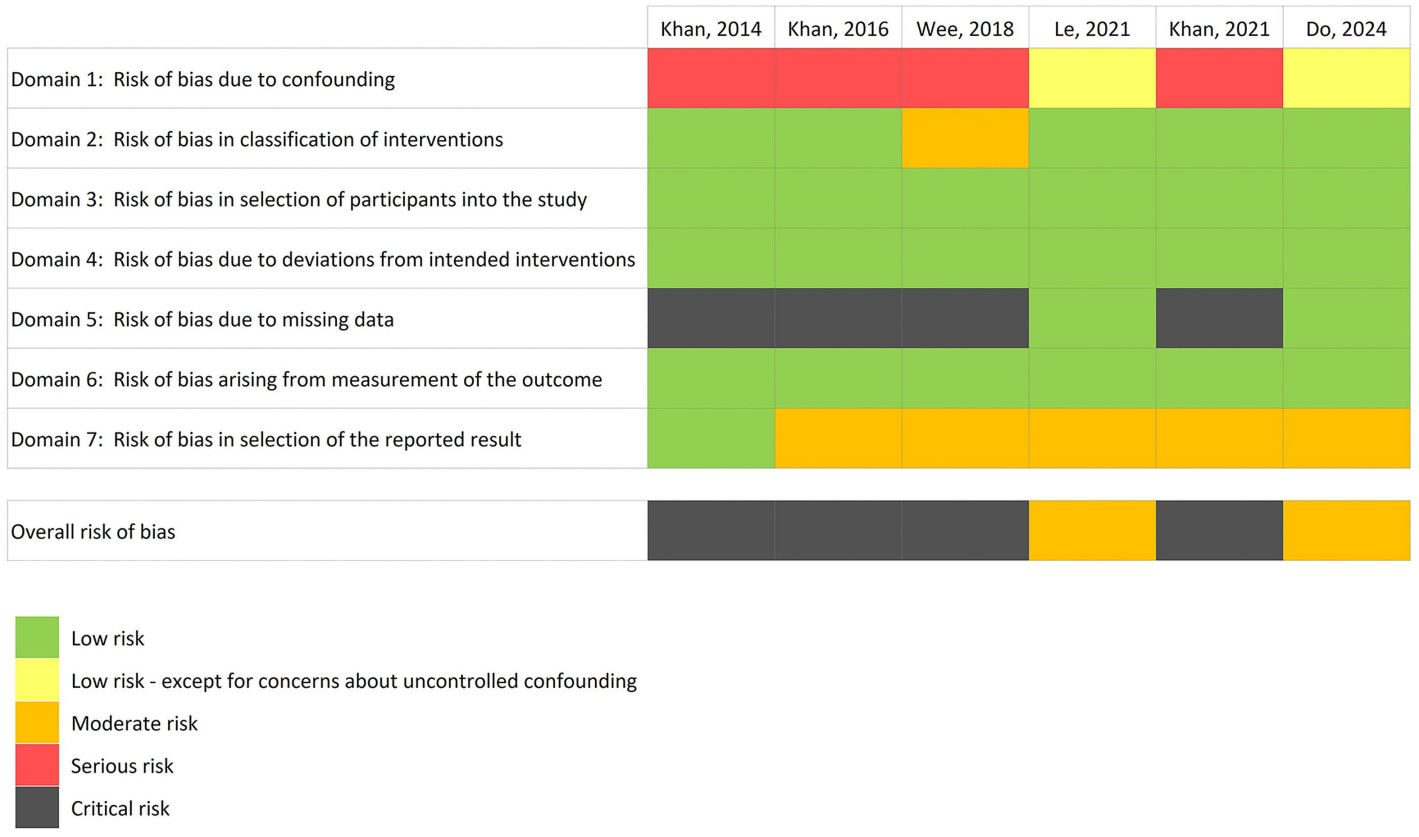
Risk of bias assessment, evaluated through ROBINS-I-V2 tool.

Since the six papers selected were based on non-randomized studies, we used the ROBINS-I-V2 tool for risk of bias assessment (Risk of Bias Tools, n.d.; Figure 3). Before evaluation, we preliminarily determined the confounding factors to be relevant for the intervention-outcome relationship under study. In detail, we considered ethnicity, body mass index (BMI), range of age, menstrual cycle phase (Proliferative/Secretive/Menstrual/Amenorrhea), endometriosis stage, use of therapies for chronic diseases, previous or concomitant use of immunosuppressive or antimicrobial agents as substantial confounding factors. Whereas age, menstrual cycle regularity (28–32 days), history of chemo-radio therapy and surgical gynecological interventions (not related to endometriosis) were assigned a comparatively lower degree of importance. Additionally, we took into consideration whether all treated patients followed the same intervention protocol.

In relation to Domain 1, the two studies from Do et al. (2024) and Le et al. (2021) showed a low level of risk of bias due to confounding factors. On the contrary, the risk of bias of the remaining four papers analyzed was assessed as serious, mainly due to the lack of information about the use of antimicrobial agents, BMI and ethnicity of patients. Consequently, the aforementioned papers also exhibited a critical risk of bias attributable to missing data (Domain 5).

Due to the lack of information about the type of oral contraceptive used, Wee et al. (2018) was assigned a moderate level of risk of bias in classification of interventions (Domain 2). All other studies showed a low risk of bias in the same domain.

The risk of bias for Domain 3 (Risk of bias in selection of participants into the study or into the analysis), Domain 4 (Risk of bias due to deviations from intended interventions) and Domain 6 (Risk of bias arising from measurement of the outcome) was found low in all the selected papers. For Domain 7 (Risk of bias in selection of the reported result), all papers were attributed a low or moderate risk of bias. In particular, the study from Khan et al. (2014), in which the outcome was measured through the assessment of Nugent score, was assigned a low level of risk since there is only one possible way in which the outcome domain can be measured. The other five papers, based on NGS techniques, have been attributes a moderate level of risk, due the existence of multiple strategies to analyze the outcome.

In consideration of these results, the overall risk of bias for Do et al. (2024) and Le et al. (2021) was categorized as moderate. Conversely, the overall risk for Khan et al. (2014), Khan et al. (2016), Wee et al. (2018), and Khan et al. (2021) was deemed critical.

### 3.4 Synthesis of results

Research studies investigating the impact of hormonal treatments on the composition of the reproductive tract microbiome in women with endometriosis reported heterogeneous findings, largely depending on therapeutic approach and methodological differences. Interventions include GnRHa treatment and contraceptive therapy (COCs and progestins), whereas methodologies include standard culture methods, Gram staining and NGS techniques, as illustrated in the following sections and in Table 2.

**Table 2:** Results of the single studies included in the review.

GnRH agonists							
Study	Samples	Methods	Statistical analysis	Endometriosis vs control	Main findings (HT vs NT in endometriosis)		Conclusions
					Endometrium	Vagina	
Khan et al. (2014)	Vaginal and endometrial	Bacterial cultures, Gram staining, Nugent scoring system	Chi-square and Kruskal-Wallis tests	↑ sub-clinical uterine infection; ↑ vaginal pH	↑ *Gardnerella*, *Escherichia coli*, and *Enterococci*; ↑ endometritis	↑ intermediate flora (Nugent 4–6); ↓ normal flora (Nugent 0–3); ↑ vaginal pH	GnRHa has a negative impact on vaginal and endometrial microbial communities, favoring the colonization of potentially pathogenic bacteria.
Khan et al. (2016)	Endometrial and cystic fluid	16S rRNA gene sequencing (V3-V4 regions)	Mann–Whitney U-test	In endometrium: ↑ *Streptococcaceae* and *Moraxellaceae;* in cystic fluids: ↑ S*treptococcaceae, Staphylococcaceae*, ↓ *Lactobacillaceae*	↑ *Streptococcacee, Staphylococcaceae* and *Enterobacteriaceae; ↓ Lactobacillaceae*		GnRHa has a negative impact on vaginal and endometrial microbial communities, favoring the colonization of potentially pathogenic bacteria.
Khan et al. (2021)	Endometrial	16S rRNA gene sequencing (V5-V6 regions)	Shannon index, Mann–Whitney U-test	↑ α-diversity	↓ α-diversity; ↑ *Gardnerella*; ↓ *Streptococcus* and *Prevotella*		GnRHa treatment negatively affects microbial diversity, favoring some potentially pathogenic bacteria while impairing others.

Oral contraceptives							
Study	Samples	Methods	Statistical analysis	Endometriosis vs control	Main findings (HT vs NT in endometriosis)		Conclusions
					Endometrium	Vagina	
Le et al. (2021)	Vaginal	16S rRNA gene sequencing (V4 region)	Faith’s PD index, weithted and unweighted UniFrac metrics, PERMANOVA	Different β-diversity; ↓ *α*-diversity; ↑ *Lactobacillus*		Different β-diversity; ↑ α-diversity; ↓ *Lactobacillus; ↑ Peptoniphilus, Dialister, Finegoldia, Prevotella* and *Ureaplasma* genera; ↑ *Actinobacteria; ↑ B/B* ratio	Oral contraceptive pill alters vaginal microbial composition prompting dysbiosis.
Do et al. (2024)	Vaginal	16S rRNA gene sequencing (V4 region)	Faith’s PD index, Bray Curtis metric, Kruskall-Wallis test			↓ α-diversity; Different *β*-diversity	Hormonal therapy has limited effect within the urogenital tract.
Wee et al. (2018)	Vaginal, cervical and endometrial	16S rRNA gene sequencing (V3 region)	Non-parametric *t*-test			*↑ Streptococcus anginosus, Streptococcus agalactiae, Bifidobacterium breve*	Hormonal therapy may favor the colonization of potentially pathogenic bacteria.

B/B ratio, Bacillota/Bacteroidota ratio.

#### 3.4.1 GnRHa—impact on endometrial microbiome

Three studies have assessed the impact of GnRHa on endometrial microbiome composition, all consistently reporting that it induces a dysbiotic state, as detailed in the following paragraphs.

Khan et al. (2014) demonstrated for the first time that intra-uterine microbial colonization, considered as sub-clinical uterine infection, was significantly higher in women with endometriosis than in control ones. The authors also evaluated the risk of intrauterine bacterial colonization and concurrent endometritis, hypothesizing that this risk may be further increased in women under GnRHa therapy. Using standard culture methods and Gram staining of endometrial samples, they confirmed their hypothesis showing that GnRHa treatment (4–6 months) associated with increased uterine colonization by pathogenic microbes as *Gardnerella* and *Escherichia coli* in control patients, and *Gardnerella*, *Enterococci* and *Escherichia coli* in endometriosis women (*p* ≤ 0.05). Although there was no significance difference in the occurrence of acute endometritis between women with and without endometriosis, its frequency in GnRHa-treated groups was higher in both control and endometriosis patients compared with untreated groups (*p* = 0.003 for control group, *p* = 0.001 for endometriosis group).

In a later study, Khan et al. (2016) further investigated the microbial community in female reproductive tract through the employment of NGS approach. By performing 16S rRNA V3–V4 regions sequencing on endometrial swabs, the authors evaluated the rate of microbial colonization in the intrauterine environment and in the cystic fluid of women affected or not by endometriosis, further grouped based on GnRHa treatment (1.88 mg IM per month, for 4–6 months). Firstly, they observed a higher percentage of two microbial families, *Streptococcaceae* and *Moraxellaceae* in endometriosis patients compared with unaffected women. A similar trend was detected in cystic fluids, where abundances of *Streptococcaceae* (*p* < 0.01) and *Staphylococcaceae* (*p* < 0.05) were significantly increased and *Lactobacillaceae* (*p* < 0.01) reduced in endometriomas compared with non-endometriotic cysts. As regards to GnRHa treatment, in control women it promoted a higher *Staphylococcaceae* (*p* < 0.05) uterine colonization. Whereas in endometriosis patients, GnRHa therapy induced a decrease in the content of *Lactobacillaceae* (*p* < 0.01) and an increased abundance of *Streptococcaceae*, *Staphylococcaceae* and *Enterobacteriaceae* (*p* < 0.05) in the endometrium.

These results were partially confirmed by a more recent study conducted by Khan et al. (2021), in which they evaluated the effect of GnRHa (1.88 mg IM per month, for 3 months) and levofloxacin, as single or combined therapy, in control women or patients diagnosed with endometriosis. By using 16S rRNA metagenome assay (V5–V6 regions sequencing), the authors reported a reduced *α*-diversity in the endometrium of GnRHa-treated endometriosis women (*p* = 0.001), with higher abundance of *Gardnerella.* Contrarily to the evidence published in Khan et al. (2016), the administration of GnRHa therapy to endometriosis patients associated with a lower prevalence of *Streptococcus* along with *Prevotella* genera compared to untreated women. In healthy controls, however, α-diversity remained unaffected by treatment (*p* < 0.05).

In these three studies endometrial samples were collected by means of seed swabs. Despite the authors stated that the risk of cross contamination was minimized avoiding any contact with vaginal walls, no controls were implemented.

#### 3.4.2 GnRHa—impact on vaginal microbiome

Some evidence regarding the vaginal environment have been provided by Khan et al. (2014). The authors took into consideration the occurrence of BV, assessed through a modified Nugent scoring system based on *Lactobacillus* spp., *Gardnerella vaginalis* and *Mobiluncus* spp. counts. They observed that, in women with endometriosis, GnRHa treatment (4–6 months) promotes an increase of intermediate vaginal microflora (*p* = 0.05) and a concomitant decrease of normal microflora (*p* = 0.007). A similar result was found in control group, despite not statistically significant. Another parameter that associates with the microbial milieu is vaginal pH. Indeed, changes of this factor associate with variations of the bacterial community, in particular a shift toward an alkaline environment (pH ≥ 4.5) allows for the outgrowth of potentially pathogenic organisms (Landolt et al., 2025). In the same study, Khan et al. observed a baseline difference in vaginal pH between women with endometriosis and control women, with endometriosis group most frequently harboring pH ≥ 4.5 (79.3% versus 58.4%, respectively, *p* < 0.03). In women treated with GnRHa, vaginal pH was increased (≥4.5) in both control women (*p* = 0.004) and in women with endometriosis (*p* = 0.03), further underlying a pejorative effect of the therapy on vaginal microbiome.

#### 3.4.3 Oral contraceptives—impact on cervical/vaginal microbiome

Concerning oral contraceptives (OCs) therapy, including COCs and progestins, three main studies have described their effect on the microbiome of the reproductive tract in endometriosis patients.

In one study, Le et al. (2021) analyzed the putative association of microbial dynamics in patients undergoing surgical intervention for endometriosis or benign uterine or ovarian indications. The analysis was performed through NGS technology (16S rRNA gene sequencing, V4 region). The authors reported that COCs (1 mg of norethindrone and 35 micrograms of ethinyl estradiol) significantly altered gut and vaginal microbiota. Specifically, in patients not using COCs, the authors showed that the *β*-diversity in both sites (gut/vagina) was different in endometriosis patients compared to control subjects. Moreover, a correlation between disease state and the use of COCs emerged, with a significant difference observed in the vaginal bacterial communities of treated vs. not treated endometriosis patients, measured as β-diversity (unweighted R^2^ = 0.01, *p* = 0.06, weighted R^2^ = 0.02, *p* = 0.01). In addition, comparing endometriosis and control patients, in the absence of COCs, a reduced bacterial *α*-diversity was found, with higher levels of *Lactobacillus* in the first group. In endometriosis women, treatment with COCs resulted in a higher bacterial richness in the vaginal tract, likely due to the decreased abundance of *Lactobacillus* and increase of *Peptoniphilus*, *Dialister*, *Finegoldia, Prevotella* and *Ureaplasma* genera, both at the day of surgery (DOS) and post-surgical intervention (PSI). Importantly, upon COCs administration, *Actinobacteria* were increased, as well as the *Bacillota/Bacteroidota* ratio. These data are suggestive of vaginal dysbiosis, indicating that endometriotic lesions alter both the abundance and the composition of bacterial species in the vaginal tract, and that these alterations are worsened by the use of COCs.

A subsequent study developed by the same research group further investigated this topic in a cohort of 25 endometriosis patients, reporting partially conflicting results (Do et al., 2024). The types of treatment prescribed were heterogeneous: Nortrel 1/35 (20 patients), Lo Loestrin Fe 10 μg (1 patient), Norethindrone acetate 5 mg (1 patient), Seasonique 0.15–0.03 and 0.01 mg (1 patient), Lupron (1 patient) and Norgestimate/ethinyl estradiol (1 patient). However, the analysis, performed through NGS technology (16S rRNA gene sequencing, V4 region), was conducted without stratifying for this variable. They observed that vaginal samples of treated women had reduced α-diversity compared to not treated ones (*p* = 0.01) at PSI. Whereas, at DOS, no differences were reported. In concordance with their previous study, the authors assessed that the use of hormonal therapy promotes the establishment of a different bacterial community in endometriosis patients, regardless the time points analyzed (*p* = 0.015 at DOS; *p* = 0.005 at PSI).

The study from Wee et al. (2018) was designed to evaluate the association between the fertility status and the composition of reproductive tract microbiota, considering vaginal, cervical and endometrial districts. Swabs were used to sample vaginal and cervical tracts, and curettes for uterine cavity. The analysis was performed through NGS technology (16S rRNA gene sequencing, V3 region). Among the enrolled patients, 12 participants had a history of endometriosis, equally distributed in the control fertile group (*n* = 6) and in the case infertile group (*n* = 6). Among endometriosis patients, 4 assumed OCs (unspecified formulations), 4 were not under treatment, and the last 4 were using intrauterine device (IUD) as contraceptive method. Despite the authors focused on the putative differences between fertile and infertile subjects, the data included in the paper allow to make some considerations relatively to the effect of OCs on the microbiome profile of patients affected by endometriosis. As expected, it is possible to observe a concordance in bacterial composition between cervical and vaginal samples. Endometriosis patients not using OCs were characterized by a predominance of bacteria from the *Lactobacillus* genus. Specifically, *Lactobacillus iners* was the most abundant species in two participants, *Lactobacillus jensenii* in one, and *Lactobacillus crispatus* along with *Gardnerella vaginalis* in the last. In contrast, among the four patients receiving OCs treatment, while two were dominated by *Lactobacillus* genus (*Lactobacillus crispatus* in one and *Lactobacillus iners* in the other), the remaining two showed a higher abundance of *Streptococcus* genus, *Streptococcus anginosus* in one case, and *Streptococcus agalactiae* accompanied by *Bifidobacterium breve* in the other. Unfortunately, the paucity of samples (three for women with endometriosis) precludes the possibility to draw assumptions about the endometrial microbiome.

## 4 Discussion

### 4.1 Principal findings and comparison with existing literature

Endometriosis is a common gynecological disorder affecting around 10% of pre-menopausal women worldwide (Li R. et al., 2025). This condition is the leading cause of chronic pelvic pain, from adolescence to adulthood, and is often associated with infertility. In both adult and young patients, the recommended therapy includes oral hormonal administration of COCs or progestins only pills, and, as second option, GnRH agonists. Therapy must be personalized based on the peculiar characteristics of the patients, the presence of comorbidities and future reproductive aspirations.

Increasing evidence pinpoint the microbiome of the reproductive tract as a crucial determinant of the development and progression of endometriosis. In line with this view, lower richness and *α*-diversity of cervical microbiome was found in patients with more severe symptoms and infertility, suggesting that a more diverse microbial environment, with prevalence of beneficial species, might favor better clinical outcomes (Chang et al., 2022).

In partial accordance with these findings, the studies included in this systematic review evidenced a different microbial profile in patients with endometriosis compared with not affected women. Indeed, the presence of endometriotic lesions results accompanied by sub-clinical uterine infections, increased amount of *Streptococcaceae* and *Moraxellaceae* and augmented α-diversity in the endometrium (Khan et al., 2014, 2016, 2021). In women with endometriosis, Khan et al. registered an increased vaginal pH, suggestive of a dysbiotic state (Khan et al., 2014), and a decreased abundance of *Lactobacillaceae* in cystic fluids (Khan et al., 2016). Conversely, Le et al. observed a reduced α-diversity along with a higher abundance of *Lactobacillus,* indicative of a healthy balanced vaginal microbiome in endometriosis patients. These discrepancies might be partially explained by the different types of samples analyzed, vaginal swab vs. cystic fluids, and by the ethnicity of the study populations, known to have an impact on microbial profiles (Wei et al., 2024). Another crucial aspect which should be taken into consideration relies on the fact that both studies did not specify the dominant *Lactobacillus* specie, and this information is noteworthy since it is known that different species characterize different types of vaginal ecosystems (Zhu et al., 2022). Precisely, *Lactobacillus crispatus* provides protection against pathogens, whereas *Lactobacillus iners* and *Lactobacillus gasseri* often co-occurs with dysbiosis-associated microbes (De Seta et al., 2019).

**Figure 4:**
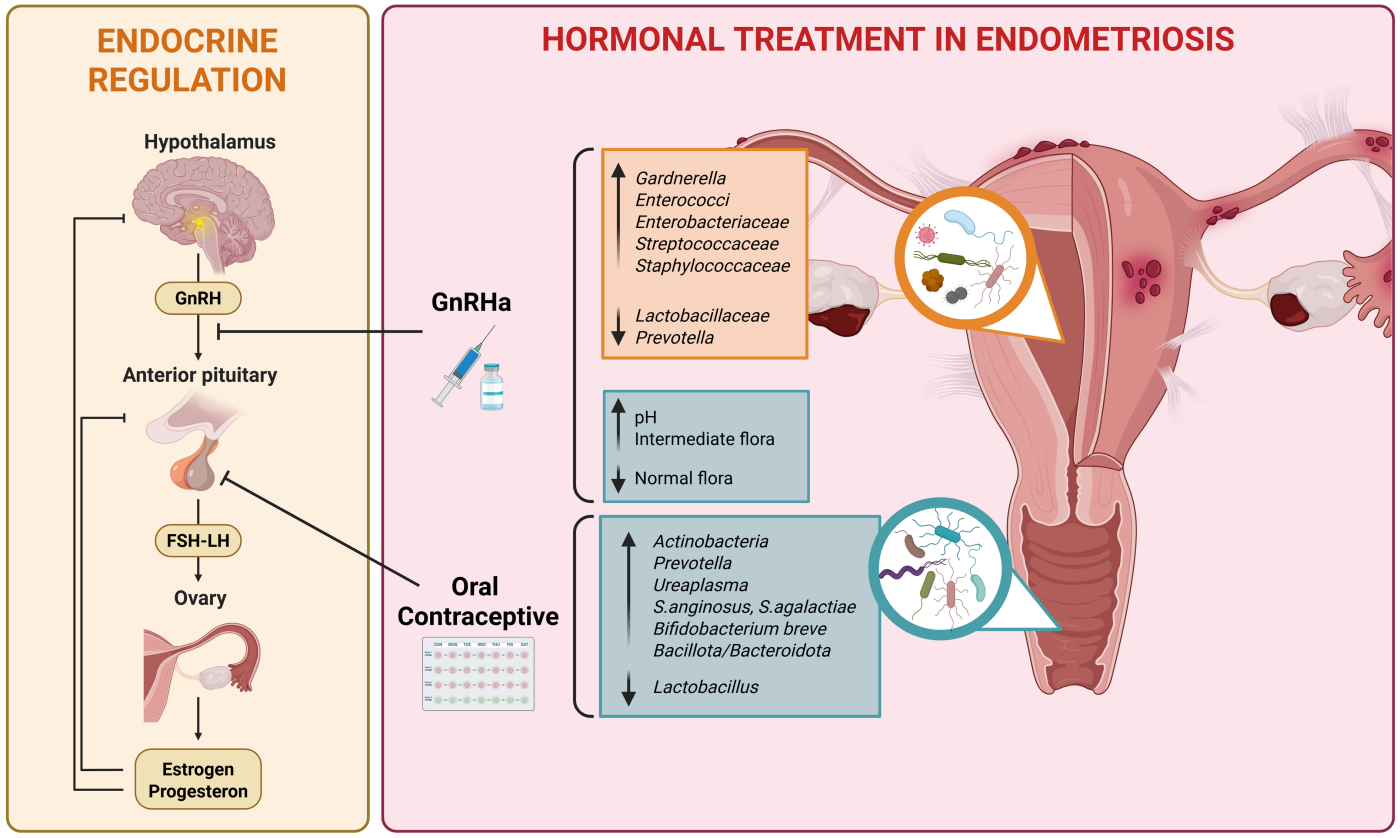
The illustration summarizes the endocrine regulation of the hypothalamic–pituitary–ovarian axis, in which hypothalamic gonadotropin-releasing hormone (GnRH) stimulates the anterior pituitary to secrete follicle-stimulating hormone (FSH) and luteinizing hormone (LH), thereby regulating ovarian estrogen and progesteron production (left). The right panel depicts the sites of action of GnRHa and oral contraceptives, which interfere with this axis and modify systemic sex-hormone signaling. Hormonal treatment in endometriosis leads to a hypo-estrogenic environment, which may associate with modifications of vaginal (blue) and endometrial (orange) milieu when compared with untreated affected women. These alterations include changes in vaginal pH and shift in microbial composition. *S. anginosus*, *Streptococcus anginosus*; *S. agalactiae*, *Streptococcus agalactiae.* Created in https://BioRender.com.

In support of the hypothesis that endometriosis is associated with a microbial disequilibrium in the reproductive tract, the recently published review from Wang et al. highlighted the correlation between microbial communities and the onset of the disease, discussing the multiple mechanisms behind, which include the modulation of inflammatory/immune responses and the regulation of estrogen metabolism (Wang et al., 2025). For instance, in preclinical studies it has been observed that the inoculation of the pathogenic specie *Fusobacterium nucleatum* increased the numbers and weights of endometriotic lesions in mice, which were effectively reduced by antibiotic treatment (Muraoka et al., 2023). In addition, it has been assessed that BV-associated bacteria (*Gardnerella vaginalis, Prevotella bivia* and *Mobiluncus* spp.) and *Lactobacillus* spp. depletion in the cervico-vaginal microbiome associate with endometriosis (Uzuner et al., 2023). In this context, Khan et al. unveiled that the use of GnRHa in women with endometriosis further alters vaginal flora and promotes the establishment of an alkaline milieu, ideal substrate for the proliferation of potentially pathogenic bacteria (Khan et al., 2014; Figure 4). The treatment with GnRHa, by establishing a hypo-estrogenic state along with a reduced anti-microbial peptides release, may promote the growth of potentially pathogenic microbes in vaginal and endometrial districts.

A similar effect is ascribable to the administration of OCs, which reduces vaginal *Lactobacillus* and favors the colonization of microorganisms as *Prevotella, Streptococcus* and *Ureaplasma* (Figure 4). Such dysbiotic environment has the potential to contribute to the discomfort experienced by affected women. Importantly, endometriosis patients under OCs therapy, had a higher *Bacillota/Bacteroidota* ratio. *β*-glucuronidase-producing *Bacillota* are known to deconjugate estrogen metabolites elevating bioactive estrogen levels in the serum (Elkafas et al., 2022). The increase of vaginal *Bacillota* in endometriosis women treated with OCs may lead to the establishment of a therapeutic paradox cycle, in which the administration of hormonal therapy leads to an hyper-estrogenic condition, overall potentially interfering with the efficacy of the treatment (Li Z. et al., 2025; Wang and Wang, 2025).

It is noteworthy that the reproductive microbial profile can exert a significant influence on fertility, as well as the potential outcomes of future reproductive plans (Venneri et al., 2022). This association is supported by a recent meta-analysis which took into consideration 15 studies and evidenced a correlation between low abundance of vaginal *Lactobacillus* and female infertility (Hong et al., 2020). Accordingly, other authors posed the alteration of the vaginal microbial homeostasis as a risk factor for pregnancy related complications, like preterm delivery (<37 weeks) and late miscarriage, and a negative predictor for *in vitro* fertilization (IVF) success in terms of pregnancy rates (Bernabeu et al., 2019; Venneri et al., 2022). Increasing evidence are emerging indicating the beneficial effect of oral probiotics and specific dietary habits on the vaginal microbiome (Noormohammadi et al., 2022; Djusse et al., 2025; Udjianto et al., 2025). In particular, a study is exploring whether the administration of oral probiotics could restore the vaginal environment increasing the abundance of beneficial *Lactobacillus* species in IVF patients, thereby improving their outcomes (van Haren et al., 2025).

Similarly to the vaginal environment, an endometrial microbiota not dominated by *Lactobacilli* but colonized by *Bifidobacterium*, *Gardnerella*, *Staphylococcus* and *Streptococcus* is associated with adverse outcomes in IVF procedures (Moreno et al., 2022; Xiao et al., 2024). In accordance with these data and with the observations raised from the present review, a recent study evidenced that the use of a GnRHa protocol for endometrial preparation prior to embryo transfer in infertile endometriosis patients associated with *Staphylococcus* and *Escherichia*-*Shigella* colonization, and with worse obstetrics outcomes as miscarriage, cesarean delivery, and hypertensive disorders, compared to other interventions (Zhang et al., 2025). Given these observations, the choice of hormonal therapy for endometriosis needs to be discussed with the patient, who should be adequately counseled on the protection of future fertility.

### 4.2 Strengths and limitations

The strengths of this systematic review include its rigorous methodology and comprehensiveness in cataloging the existing evidence. Additionally, it allowed to define and highlight important confounding factors which are often overlooked in studies regarding the reproductive microbiome. The included papers regarding the effect of OCs on vaginal microbiome adopted the same analytical method to measure the outcomes (NGS), giving us the opportunity to draw considerations based on homogeneous data. Nonetheless, the depth of microbial characterization varied among the selected investigations, with authors reporting changes at different taxonomic levels (e.g., family, genus or species). Even species that belong to the same family or genus can differ in terms of their pathogenicity or beneficial role. For this reason, it is recommended that future investigations use NGS methods to precisely characterize the microbiome and determine the composition at species level.

A limitation of the present work relies on the lack of consideration of several confounding factors in the selected studies, as evidenced through risk of bias assessment performed with ROBINS-I V2 tool. For instance, four papers did not register any previous treatment with either immunosuppressing or antimicrobial agents for enrolled patients. This issue has been considered a bias of great importance since the recent use of antibiotics may clearly impact the outcomes. In addition, probably due to the limited sample size, none of the investigations reported a stratification of the patients according to patients age, ethnicity, menstrual cycle phase or BMI. Conversely, these features are known factors influencing the microbiome of the reproductive tract (Garg et al., 2023; Kumar et al., 2023; Dubé-Zinatelli et al., 2024; King et al., 2024; Wei et al., 2024; Landolt et al., 2025). Moreover, another factor which has been registered in all the studies but not taken into account for patient stratification is the stage of endometriosis. In this regard, recent findings have identified the vaginal microbiome as a potential tool for disease staging. For instance, Perrotta et al. (2020) reported that patients with ASRM stages III-IV exhibited a significantly different microbial composition and an enrichment for *Anaerococcus* during the menstrual phase when compared with stages I-II.

Considering the studies based on the use of OCs, the conclusions may be affected by the heterogeneous types of adopted formulations. Most of the results based on the use of COCs therapy, however, Do et al. also used GnRHa and progestin only (norethindrone acetate) medications, even if in only two participants, and Wee et al. did not specify the therapeutic regimen. There are minimal and controversial published data indicating whether COCs or progestin only therapy could exert different effects on the vaginal microbial environment (Vodstrcil et al., 2013; Ratten et al., 2021; Bakus et al., 2023), nonetheless this aspect might not be underestimated and further studies are mandatory to better understand their impact on the microbiome of endometriosis patients.

Importantly, when interpreting the results of the four studies analyzing endometrial and cervical samples, it is crucial to consider the risk of cross-contamination with the vaginal environment. Although sample collection was performed in accordance with protocols aimed at minimizing this risk, no contamination controls were applied to strengthen the robustness of the conclusions.

Ultimately, due to the unfeasibility of conducting a meta-analysis, the implementation of specific analytical tools for the evaluation of the certainty of evidence was not possible. Nonetheless, some considerations should be raised on the limited strength of the evidence emerged from the present systematic review. Indeed, the interpretation of the overall results require caution, given critical risk of bias, inconsistency across studies in the characteristics of the populations, interventions, methods, or outcomes measurement, and imprecision deriving from the low number of participants.

### 4.3 Conclusions and implications

To the best of our knowledge, this is the first systematic review which highlights the up-to-date evidence on the effect of hormonal therapy on the taxonomic composition of microbial environment of vaginal and uterine cavities in women affected by endometriosis. Despite the aforementioned limitations, the overall results suggest that both the administration of GnRHa and OCs might negatively affect the reproductive microbiome, establishing a milieu dominated by potentially harmful bacteria (Figure 4).

Since the microbial profile is tightly linked to the efficacy of the therapy and to the global health status of women, these considerations may be relevant for a better clinical management of patients. Despite our observations suffer of the criticisms due to heterogeneity of the analyzed studies, they lay the foundations for a future debate among clinicians on the routine screening of microbial dynamics alongside treatment. Similarly, in light of the findings raised from this review, it would be advisable to better investigate on the beneficial effect of probiotics supplementation or an appropriate dietary regimen to further improve patients’ outcomes. Moreover, as endometriosis onset often occurs at young age, the implications of hormonal therapy-associated dysbiosis might be considered in regard to fertility and reproductive health.

Importantly, our work unveils significant factors that need to be considered when designing investigations on reproductive microbiome in endometriosis. For instance, it will be recommended to define inclusion and exclusion criteria taking into account the recent use of antimicrobial or immunosuppressive agents and the type of intervention. Moreover, it will be mandatory to stratify the analyses by age, ethnicity, BMI, stage of endometriosis and menstrual cycle phase. To grant the appropriate homogeneity of the results, standardization of the microbiome assessment methodology is strongly suggested, in particular the use of NGS technology should be preferred to achieve species-level classification.

## Data Availability

The original contributions presented in the study are included in the article/Supplementary material, further inquiries can be directed to the corresponding authors.
