# Supplementary material for: Impact of hormonal treatments for endometriosis on the reproductive microbiome: a systematic review
Source: Front Microbiol. 2026 Feb 12;17:1755725. doi: 10.3389/fmicb.2026.1755725 (PMC12936021; doi:10.3389/fmicb.2026.1755725)
Supplement: Supplementary file 1 [file Table_1.doc]

| **Database** | **Search strategy** |
| --- | --- |
| **Pubmed** | (endometriosis) AND (oral hormonal therapy) AND (bacterial vaginosis) OR (vaginal dysbiosis) |
|  | (endometriosis) AND (hormonal therapy) AND (bacterial vaginosis) OR (vaginal dysbiosis) |
|  | (endometriosis) AND ((vaginal microbiome) OR (endometrial flora) OR (bacterial vaginosis) OR (vaginal dysbiosis)) AND ((GnRH agonist) OR (progestin) OR (combined oral contraceptive) OR (oral hormonal therapy)) |
|  | ((bacterial vaginosis) OR (dysbiosis)) AND (endometriosis hormonal therapy) |
| **Scopus** | (vaginal dysbiosis OR vaginosis) AND endometriosis AND endometriosis AND hormonal AND therapy |
|  | ( endometriosis ) AND ( ( vaginal microbiome ) OR ( endometrial flora ) OR ( bacterial vaginosis ) OR ( vaginal dysbiosis ) ) AND ( ( GnRH agonist ) OR ( progestin ) OR ( combined oral contraceptive ) OR ( oral hormonal therapy ) ) AND PUBYEAR > 1999 AND PUBYEAR < 2027 AND ( LIMIT-TO ( DOCTYPE , "ar" ) ) AND ( LIMIT-TO ( LANGUAGE , "English" ) ) |
|  | endometriosis AND hormonal therapy AND vaginal dysbiosis OR bacterial vaginosis |
| **Scholar** | (endometriosis) AND (hormonal therapy) AND (bacterial vaginosis) |
|  | (endometriosis) AND ((vaginal microbiome) OR (endometrial flora) OR (bacterial vaginosis) OR (vaginal dysbiosis)) AND ((GnRH agonist) OR (progestin) OR (combined oral contraceptive) OR (oral hormonal therapy)) |
|  | (endometriosis) AND (hormonal therapy) AND (vaginal dysbiosis) |

Supplementary Table 1. The search strategy used in all databases.
